# Bilateral lung transplantation for pediatric pulmonary arterial hypertension: perioperative management and one-year follow-up

**DOI:** 10.3389/fcvm.2023.1193326

**Published:** 2023-06-27

**Authors:** Thomas Jack, Julia Carlens, Franziska Diekmann, Hosan Hasan, Philippe Chouvarine, Nicolaus Schwerk, Carsten Müller, Ivonne Wieland, Igor Tudorache, Gregor Warnecke, Murat Avsar, Alexander Horke, Fabio Ius, Dmitry Bobylev, Georg Hansmann

**Affiliations:** ^1^ Department of Pediatric Cardiology and Critical Care, Hannover Medical School, Hannover, Germany; ^2^ European Pediatric Pulmonary Vascular Disease Network, Berlin, Germany; ^3^ Department of Pediatric Pulmonology, Allergology and Neonatology, Hannover Medical School, Hannover, Germany; ^4^ Department of Pediatric Hematology and Oncology, Hannover Medical School, Hannover, Germany; ^5^ Department of Cardiac Surgery, University Hospital of Zürich, Zürich, Switzerland; ^6^ Department of Cardiac Surgery, Ruprecht-Karls-University, Heidelberg, Germany; ^7^ Department of Cardiothoracic, Transplantation and Vascular Surgery, Hannover Medical School, Hannover, Germany

**Keywords:** pediatric, children, lung transplantation, pulmonary arterial hypertension, extracorporeal membrane oxygenation (ECMO), awake ECMO

## Abstract

**Background:**

Bilateral lung transplantation (LuTx) remains the only established treatment for children with end-stage pulmonary arterial hypertension (PAH). Although PAH is the second most common indication for LuTx, little is known about optimal perioperative management and midterm clinical outcomes.

**Methods:**

Prospective observational study on consecutive children with PAH who underwent LuTx with scheduled postoperative VA-ECMO support at Hannover Medical School from December 2013 to June 2020.

**Results:**

Twelve patients with PAH underwent LuTx (mean age 11.9 years; age range 1.9–17.8). Underlying diagnoses included idiopathic (*n* = 4) or heritable PAH (*n* = 4), PAH associated with congenital heart disease (*n* = 2), pulmonary veno-occlusive disease (*n* = 1), and pulmonary capillary hemangiomatosis (*n* = 1). The mean waiting time was 58.5 days (range 1–220d). Three patients were bridged to LuTx on VA-ECMO. Intraoperative VA-ECMO/cardiopulmonary bypass was applied and VA-ECMO was continued postoperatively in all patients (mean ECMO-duration 185 h; range 73–363 h; early extubation). The median postoperative ventilation time was 28 h (range 17–145 h). Echocardiographic conventional and strain analysis showed that 12 months after LuTx, all patients had normal biventricular systolic function. All PAH patients are alive 2 years after LuTx (median follow-up 53 months, range 26–104 months).

**Conclusion:**

LuTx in children with end-stage PAH resulted in excellent midterm outcomes (100% survival 2 years post-LuTx). Postoperative VA-ECMO facilitates early extubation with rapid gain of allograft function and sustained biventricular reverse-remodeling and systolic function after RV pressure unloading and LV volume loading.

## 1. Introduction

For children with severe pulmonary arterial hypertension (PAH) (1–4) who are not responsive to pharmacotherapy, bilateral lung transplantation (LuTx) remains the only established treatment option with proven survival benefit (5). Although PAH is the second most common indication for LuTx in children (6), data on the best perioperative management (including ECMO) (7), on pre- and postoperative cardiac function, and on mid-/long-term outcomes after LuTx are lacking.

Historically, combined heart-lung-transplantation (HLTx) had been the favored treatment option for children with treatment-resistant, end-stage pulmonary vascular disease (PVD), PAH, and right ventricular (RV) failure; however, HLTx continues to be limited by the availability of heart-lung-blocks for transplantation. According to registry analyses (ISHLT, UNOS), isolated bilateral LuTx for PAH results in long-term outcomes and survival that are similar to other pediatric LuTx-indications (6, 8, 9). Recently, we demonstrated full recovery of systolic RV function within two months after LuTx, irrespective of the cardiac compromise pre-LuTx, in a prospective study on children with PAH undergoing LuTx (10) (group 1 pulmonary hypertension, WSPH 2018; Supplementary Table S1) (3), suggesting that LuTx should be preferred over heart-lung-transplantation even with severe RV dysfunction.

Early complications following LuTx for PAH are often attributed to the increased left ventricular (LV) preload in the setting of chronic LV deconditioning, leading to LV diastolic dysfunction, left atrial hypertension, consecutive severe pulmonary edema, and primary graft dysfunction. Thus, we introduced the concept of default peri-/post-transplant veno-arterial extracorporeal membrane oxygenation (VA-ECMO) in adult patients with severe PAH undergoing LuTx at our center in 2010 (11).

In earlier decades, the use of ECMO pre- or post-LuTx in children was associated with higher complication rates and poorer outcomes compared to LuTx without the need for ECMO support (12). Application of awake-ECMO as bridge-to-transplantation, not requiring any mechanical ventilation and sedation, greatly improved outcomes after LuTx compared to mechanical ventilation/sedation plus ECMO in adults (13, 14); meanwhile, awake-ECMO has been extended to pediatric patients of all age groups and other indications for ECMO than PAH (15, 16).

Data on perioperative management using scheduled (default) ECMO support for pediatric patients with PAH (including children <2 years of age), and its relation to recovery of heart-lung function and mid- to long-term clinical outcomes, have not been systematically analyzed. In this study, we present data on consecutive PAH patients <18 years of age who underwent LuTx with default postoperative VA-ECMO and intention of early extubation at our center from December 2013 to June 2020.

## 2. Methods

### 2.1. Patient population

We conducted a prospective observational study of 12 consecutive children with severe PAH who underwent bilateral LuTx at Hannover Medical School between December 2013 and June 2020 (Table 1 and Supplementary Tables S1, S2). The patients had at least a 12-months-post-LuTx diagnostic follow-up with transthoracic echocardiography and pulmonary function testing. Survival was analyzed both 12 and 24 months post-LuTx, until September 2022. Two excluded patients are described in the Supplementary Material. We defined PAH according to the World Symposium of PH (WSPH, Nice 2018) (3, 17) (Supplementary Table S1**)**: mPAP >20 mmHg, PAWP ≤15 mm Hg, and pulmonary vascular resistance (PVR) index ≥3 WU·m^2^ when >3 months old, at sea level (2). We only enrolled children with PAH-LuTx and excluded LuTx patients in WSPH diagnosis group 2–5 PH. We calculated the European Pediatric Pulmonary Vascular Disease Network (EPPVDN) pediatric PH risk score (18), consisting of 17 clinical, echocardiographic, and hemodynamic variables, to assess the patients' condition prior to transplant (Table 1).

**Table 1:** Patient characteristics.

Patients #1–12	At LuTx *N* = 12
Demographics	
Age – years	11.9 ± 1.4 (1.9–17.8)
Sex, Female – *n* (%)	9 (75%)
Height – m	1.5 ± 0.1 (0.8–1.8)
Weight – kg	35.2 ± 4.3 (8.2–58.0)
BSA – m^2^	1.2 ± 0.1 (0.4–1.7)
Clinical diagnosis	
PH Group 1 – *n*	
1.1 IPAH	4
1.2 HPAH (BMPR2, *n* = 3; TBX4, *n* = 1)	4
1.4.4 PAH-CHD	2
1.6 PVOD/PCH	2
Functional status pre-LuTx	
WHO Functional Class	3.7 ± 0.1
6 MWD (0 m for ECMO)^a^ – m, *n* = 11	210 ± 56
6 MWD (last before LuTx) – m, *n* = 11	276 ± 50
NT-proBNP—ng/L	3,380.7 ± 1,106.0
Invasive hemodynamics pre-LuTx	
mRAP – mm Hg, *n* = 11	9.4 ± 1.3
RVEDP – mm Hg, *n* = 10	12.7 ± 1.0
mPAP/mSAP, *n* = 11	1.2 ± 0.1
PVRi – WU·m^2^, *n* = 11	27.3 ± 2.4
PVR/SVR, *n* = 11	1.4 ± 0.2
Cardiac index (Qsi) – L/min/m^2^, *n* = 11	2.7 ± 0.2
Risk stratification (EPPVDN) pre-LuTx	
Total patients – *n*	12
Noninvasive Risk – *n*	Higher Risk – 8 Intermediate Risk – 4
Noninvasive Higher Risk Score, max. 15 (decimal)	10.3/15 (0.69 ± 0.05)
Noninvasive Lower Risk Score, max. 14 (decimal)	2.0/14 (0.14 ± 0.02)
Patients with cath 0–12 months pre-LuTx – *n*	11
Invasive Risk – *n*	Higher Risk – 7 Intermediate Risk – 4
Invasive Higher Risk Score, max. 21 (decimal)	13.4/21 (0.63 ± 0.05)
Invasive Lower Risk Score, max. 20 (decimal)	2.9/20 (0.15 ± 0.02)
Lung function pre-LuTx	
FEV1 at the time of listing for LuTx – %, *n* = 10	69.7 ± 5.8
Pulmonary hypertension management	
PDE5i + ERA – *n*	1
PDE5i + ERA + i.v. epoprostenol – *n*	2
PDE5i + ERA + i.v. iloprost – *n*	1
PDE5i + ERA + i.v. treprostinil – *n*	3
PDE5i + ERA + inhaled iloprost – *n*	3
ERA + RIO + i.v. treprostinil – *n*	1
PDE5i + ERA + SEL + repetitive levosimendan – *n*	1
VA-ECMO pre-LuTx – *n*	3
VA-ECMO duration pre-LuTx – hours, *n* = 3	30 (22–292)

Values are presented as mean ± SEM. If the child had a mutation that was associated with PAH, he/she was classified as group 1.2 PH (HPAH). The indicated serum N-terminal prohormone of brain natriuretic peptide (NTproBNP) concentrations are the last measurements prior to LuTx. For risk stratification, see the new 2019 EPPVDN risk score. Of the 11 patients treated with phosphodiesterase type 5 inhibitors (PDE5i), 10 patients received sildenafil and 1 patient tadalafil. Of the 12 patients treated with endothelin receptor antagonists (ERA), 7 patients were treated with macitentan and 5 patients with bosentan. In addition to the aforementioned medication, 3 of the 12 LuTx patients were treated with amlodipine and 9/12 with spironolactone.

BSA, body surface area; cath, catheterization; CHD, congenital heart disease; EPPVDN, European Pediatric Pulmonary Vascular Disease Network; ERA, endothelin receptor antagonist; HHT, hereditary hemorrhagic telangiectasia; HPAH, hereditary PAH; IPAH, idiopathic PAH; i.v.; intravenous; LuTx, lung transplantation; mPAP, mean pulmonary arterial pressure; mRAP, mean right atrial pressure; mSAP, mean systemic arterial pressure; NT-proBNP, N-terminal pro-b-type natriuretic peptide; PAH, pulmonary arterial hypertension; PCH, pulmonary capillary hemangiomatosis; PDE5i, phosphodiesterase type 5 inhibitor; PVOD, pulmonary veno-occlusive disease; PVR, pulmonary vascular resistance; PVRi, pulmonary vascular resistance index; Qsi, systemic flow index; RIO, riociguat; RVEDP, right ventricular end-diastolic pressure; SEL, selexipag; SVR, systemic vascular resistance; WHO, World Health Organisation.

^a^ Three patients were on VA-ECMO pre-LuTx. The 6 MWD is 0 m if the patient was on VA-ECMO pre-LuTx.

### 2.2. Clinical data collection

The clinical data collection included multimodal pre-, peri-, postoperative, and follow-up data from all patients. Details on surgical management for pediatric LuTx procedures in our center have recently been published (5). Allograft function was measured by spirometry; chronic lung allograft dysfunction (CLAD) was defined according to the 2019 ISHLT consensus report (19).

### 2.3. Transthoracic echocardiography

We applied echocardiographic B-mode, M-Mode, Doppler, and ventricular 2D strain analysis (20, 21). All examinations were performed on Philipps IE33 or EPIQ CVx ultrasound machines. Images were recorded digitally and analyzed at a workstation using Intellispace Echo software (Philips Medical Systems, The Netherlands) by a single investigator. Methodological details can be found in the Supplementary Material.

### 2.4. Statistical analysis

Either the Wilcoxon signed-rank test or the paired two-tailed t-test was used to make pairwise comparisons for data collected pre-LuTx and 1-year post-LuTx depending on the outcome of the normality testing of the difference between the pairs. Samples were considered normally distributed if they passed all applied normality tests (*p*-value >0.05): D'Agostino-Pearson, Shapiro-Wilk, and Kolmogorov-Smirnov. All statistical analysis was performed in GraphPad Prism. The changes in the examined variables (Figure 3) were visualized using R and GraphPad Prism software. Data are reported as mean ± SEM, if not stated otherwise. Details on the methodology, imaging, and outcome variables can be found in the Supplementary Material and the figure legends.

### 2.5. Ethics statement

All clinical data were anonymized. Informed consent was obtained from the legal caregivers according to the principles expressed in the Declaration of Helsinki (IRB approval #2200-2014).

## 3. Results

### 3.1. Demographic and clinical characteristics at baseline

Demographic and clinical characteristics of the 12 patients with PAH undergoing LuTx are summarized in Table 1 and shown individually in Supplementary Table S2, including invasive hemodynamics and medication pre-LuTx. Age at lung transplantation ranged from 1.9 to 17.8 years (mean 11.9 years). Two patients were transplanted during the COVID-19 pandemic (2020). Six patients were under 12 years old [Lung Allocation Score (LAS) exemption], one of which had a body surface area and weight below 0.5 m^2^ and 8.5 kg. Half of the patients had failure to thrive, with a body weight below the 10th (*n* = 6) or even below the 1st (*n* = 2) percentile (cachexia). All patients were in the WSPH diagnosis group 1 PH (Table 1 and Supplementary Tables S1, S2). Underlying diagnoses included idiopathic PAH (IPAH, *n* = 4), heritable PAH (HPAH, *n* = 4), PAH associated with congenital heart disease (PAH-CHD, *n* = 2), pulmonary veno-occlusive disease (PVOD, *n* = 1), and pulmonary capillary hemangiomatosis (PCH, *n* = 1). Disease-causing heterozygous mutations affected BMPR2 (*n* = 3) and TBX4 (*n* = 1) genes. All patients were symptomatic, in WHO functional class 3 or 4, with a mean 6-minute walk distance of 210 ± 56 meters (*n* = 11) before transplantation. Mechanical circulatory support (MCS)-status, cannulation mode, cannula size, and ECMO-associated complications are displayed in Table 2.

**Table 2:** Individual patient characteristics, ECMO management, ECMO duration, and ECMO-associated complications pre and post-bilateral lung transplantation.

No	Age (years)	Sex (M/F)	Weight (kg) & percentile (%)	Height (cm)	BSA (m²)	MCS type	Venous Cannula (Size)	Location	Arterial cannula (Size)	Location	Pre-LuTx ECMO-Duration (hours)	Post-LuTx ECMO- Duration (hours)	Complications
**1**	15.0	F	43.0 (4th Perc.)	163	1.40	V-A	FemTrak (20 Fr.)	V. femoralis	NOVAPORT (15 Fr.)	A. femoralis	22	172	Vascular perforation during cannulation (Seldinger-technique, ECMO-CPR), bleeding into the mediastinal space, thoracotomy
**2**	13.2	F	40.0 (12nd Perc.)	162	1.34	V-A	FemTrak (20 Fr.)	V. femoralis	NOVAPORT (15 Fr.)	A. femoralis	292	286	Impaired distal leg perfusion with the need for embolectomy and vascular reconstruction
**3**	10.7	M	35.0 (42nd Perc.)	165	1.27	V-A	Bio-Medicus (17 Fr.)	V. femoralis	NOVAPORT (13 Fr.)	A. femoralis	0	168	Surgical revision for hemothorax on day 1
**4**	14.2	F	50.0 (34th Perc.)	168	1.53	V-A	HLS (21 Fr.)	V. femoralis	NOVAPORT (13 Fr.)	A. femoralis	0	184	None
**5**	1.9	M	8.2 (<1st Perc.)	80	0.42	V-A	Bio-Medicus (14 Fr.)	Right atrium (open chest)	Bio-Medicus (12 Fr.)	Aorta (open chest)	0	106	Intrathoracic hematoma on day 1 post-LuTx requiring surgical removal. Infarction of the arteria cerebri media, hemiparesis, complete restoration after 12 months
**6**	17.5	F	40.0 (<1st Perc.)	157	1.32	V-A	HLS (21 Fr.)	V. femoralis	NOVAPORT (13 Fr.)	A. femoralis	0	363	None
**7**	10.3	F	25.0 (3rd Perc.)	122	0.92	V-A	Bio-Medicus (15 Fr.)	V. femoralis	Bio-Medicus (12 Fr.)	A. femoralis	0	183	None
**8**	11.7	M	33.0 (15th Perc.)	144	1.15	V-A	Bio-Medicus (17 Fr.)	V. femoralis	NOVAPORT (13 Fr.)	A. femoralis	46	159	None
**9**	17.8	F	58.0 (46th Perc.)	175	1.68	V-A	Novaport (15 Fr.)	V. femoralis	NOVAPORT (15 Fr.)	A. femoralis	0	73	None
**10**	16.2	F	52.0 (24th Perc.)	164	1.54	V-A	Novaport (15 Fr.)	V. femoralis	NOVAPORT (15 Fr.)	A. femoralis	0	216	None
**11**	5.5	F	16.8 (9th Perc.)	115	0.73	V-A	Bio-Medicus (15 Fr.)	V. femoralis	Bio-Medicus (12 Fr.)	A. femoralis	0	146	Leg ischemia post ECMO-explantation requiring surgical embolectomy
**12**	8.2	F	21.8 (7th Perc.)	130	0.89	V-A	Biomedicus (15 Fr.)	V. femoralis	NOVAPORT (13 Fr.)	A. femoralis	0	163	None

BSA, body surface area; LuTx, lung transplantation; MCS, mechanical circulatory life support; V-A, veno-arterial.

### 3.2. Clinical presentation at the time of listing for lung transplantation

Preoperative echocardiography showed imminent (*n* = 9) or acute right heart failure (*n* = 3), systemic/suprasystemic right ventricular (RV) pressure (*n* = 12), systolic RV dysfunction (*n* = 12), end-systolic septal shift with left ventricular (LV) compression (*n* = 12), and pericardial effusion in different degrees. The mean serum NT-proBNP concentration before LuTx was 3,381 pg/ml (median 1,113, range 110–10,972 pg/ml; *n* = 12); of note, several patients were admitted to the hospital in critical condition with several fold higher NT-proBNP levels which then improved under therapy. At the time of LuTx, 8 patients were EPPVDN pediatric PH “higher risk”, and 4 were “intermediate risk”. The mean “non-invasive higher risk score” in the 12 patients was 10.3 (max. score 15; decimal score 0.69 ± 0.05) (Table 1). Pre-transplant lung function testing (Table 1) at the time of listing showed a mean of 69.7% (*n* = 10) predicted for FEV1 (range 43.0%–93.8%) and 72% (*n* = 10) predicted for FVC (range 40%–102%). FEV1 and FVC were markedly reduced in one patient with additional interstitial lung disease (non-specific interstitial pneumonia by biopsy) attributed to TBX4-mutation and in two patients with airway compression attributed to enlarged pulmonary arteries. The mean LAS in the six patients ≥12 years was 47.2 (range 32.2–70.5). The mean waiting time on the LuTx list was 58.5 days (range 1–220 days) for the cohort and 10.7 days (range 1–14 days) for the three patients on pre-LuTx ECMO.

### 3.3. Emergency VA-ECMO cannulation and VA-ECMO-CPR preceding LuTx

Three patients transferred to our hospital for LuTx evaluation required emergency VA-ECMO cannulation because of acute right-heart failure/pulmonary vascular crisis (Tables 1, 2 and Supplementary Table S2). Two of these patients underwent cardiopulmonary resuscitation (CPR) in our intensive care unit and rescue ECMO-cannulation (ECMO-CPR). All three patients were successfully extubated to undergo awake-pre-LuTx-ECMO, without any long-term neurological deficit.

### 3.4. Bilateral lung transplantation

All 12 patients underwent bilateral sequential LuTx. Sternum-sparing bilateral thoracotomies were performed for surgical exposure whenever possible, and peripheral VA-ECMO cannulated via the right-sided groin vessels was used for cardiopulmonary support in these patients. Clamshell thoracotomy was reserved for patients where cardiopulmonary support had to be instituted by cannulating the aorta and central veins due to the small patient size (usually patients younger than 6 years old) or the need for concomitant cardiac surgery. Two patients underwent concomitant closure of a secundum atrial septal defect (ASD) on cardiopulmonary bypass (CPB) with bicaval cannulation (patient #8) or cannulation via femoral vessels (patient #10) (during LuTx). After ASD closure, CPB was switched to VA-ECMO in patient #10 to perform LuTx. Patient #8, who underwent rescue ECMO-cannulation via femoral vessels pre-LuTx, was weaned from CPB after ASD closure, followed by LuTx on VA-ECMO. One patient (#11) underwent bilateral LuTx on CPB with peripheral cannulation, and after LuTx, CPB was switched to VA-ECMO using the same cannula. The remaining nine patients were transplanted on VA-ECMO without the use of CPB (Table 3 and Supplementary Table S3).

**Table 3:** Bilateral lung transplantation, postoperative course, and clinical follow-up in pediatric patients.

Patients #1–12	PAH patients undergoing LuTx *N *= 12
Bilateral lung transplantation	
MCS type during LuTx	VA-ECMO, *n* = 11; CPB, *n* = 1
Associated procedures in the same operation	ASD closure on CPB, *n* = 2
Operation time (cut-suture) – hours (range)	6.6 ± 0.5 (4.6–10.1)
Postoperative course after LuTx	
Post-LuTx ECMO-duration – hours (range)	185 (73–363)
Post-LuTx ventilation time on ECMO – hours	40 (17–144)
Post-LuTx ventilation time after ECMO-explantation – hours	2 (0–6)
Tracheostomy (number)	0
ICU stay post-LuTx – days	15 ± 2 (4–32)
In-hospital stay post-LuTx – days	41 ± 4 (21–62)
Clinical follow-up	
Lung function post-LuTx	
FEV1 3 months post-LuTx (%), *n* = 11	73 ± 5 (52–106)
FEV1 12 months post-LuTx (%), *n* = 11	83 ± 6 (57–125)
Impeding rejection, number of steroid pulses	2 ± 0.58 (0–5)
Survival post-LuTx – months (range; % survival)	61 (26–104; 100% survival)
Number of Re-LuTx – *n* (%)	1 (0.08%)^a^

Values are presented as mean ± SEM (range). Survival is indicated according to the end of the follow-up (September 1, 2022).

ASD, atrial septal defect; CPB, cardiopulmonary bypass; FEV1, forced expiratory volume in the first second; ICU, intensive care unit; LuTx, lung transplantation; MCS, mechanical circulatory support; VA-ECMO, veno-arterial extracorporeal membrane oxygenation.

^a^ One patient underwent Re-LuTx due to CLAD 3 31 months after initial LuTx (i.e. after the 2-year follow-up period).

### 3.5. Postoperative course after LuTx for severe PAH and RV failure

**Figure 1:**
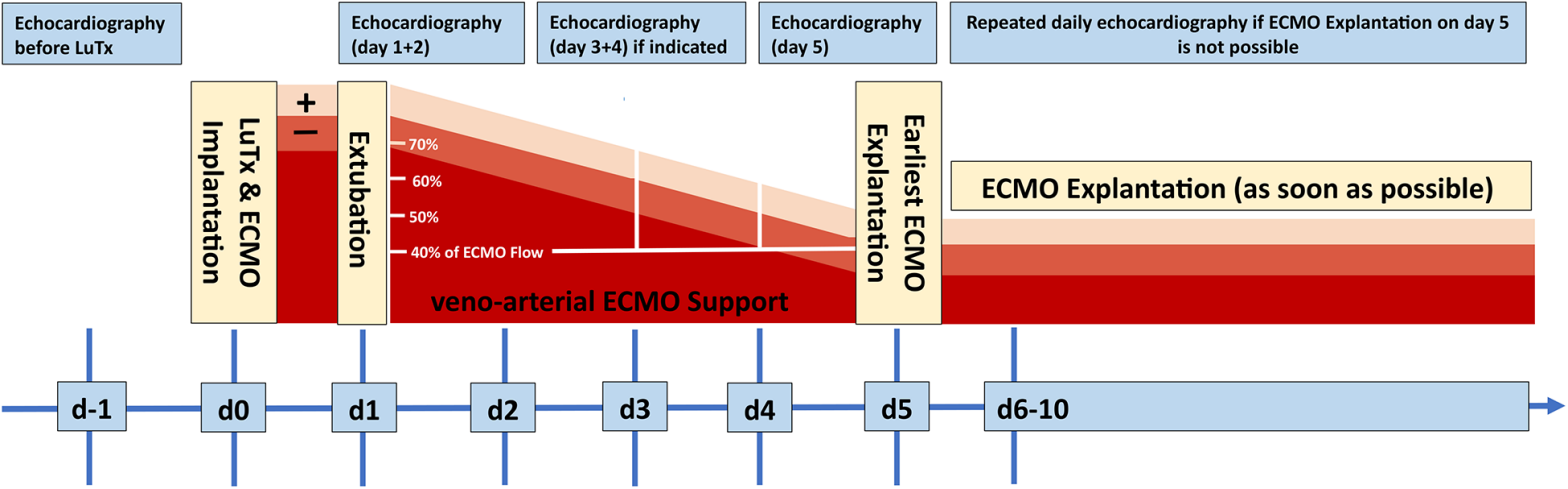
Schematic treatment and weaning algorithm of VA-ECMO treatment after LuTx. All patients were treated according to this interdisciplinary, in-house consensus standard. ECMO, extracorporeal membrane oxygenation; d, day; LuTx, lung transplantation.

**Figure 2:**
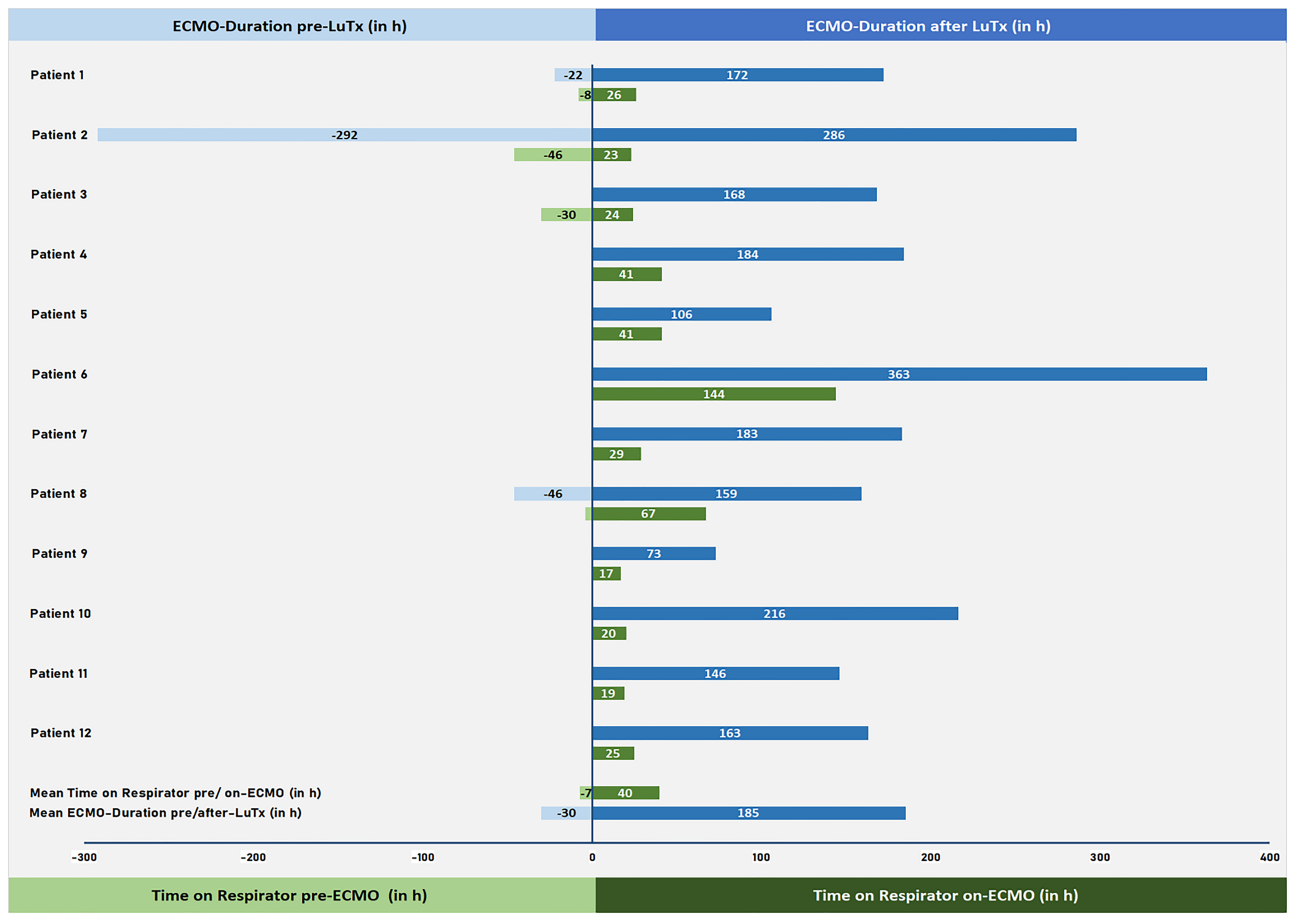
VA-ECMO duration and length of invasive mechanical ventilation pre/post-LuTx. VA-ECMO duration pre- and post-LuTx (in hours; blue) and time on respirator pre-ECMO and on-ECMO (in hours; green) are shown. VA-ECMO, veno-arterial extracorporeal membrane oxygenation; h, hours; LuTx, lung transplantation.

The mean ICU stay post-LuTx was 15 ± 2 (range 4–32) days. The average in-hospital stay post-LuTx was 41 ± 4 (range 21–62 days) (Table 3 and Supplementary Table S3). There was no perioperative (30 days post-op) mortality. The scheduled minimal duration of VA-ECMO support in our protocol is 5 days post-LuTx to assist the pressure-unloaded RV and volume-loaded LV, although two patients were weaned-off ECMO <120 h post-LuTx (Figure 1). The mean duration of post-LuTx VA-ECMO support was 185 h (range 73–363 h) [Figure 2, details in (5)]. Except for patient #5, who was the smallest child at 8.2 kg, all patients were cannulated in the groin via femoral vein and artery (Table 2). Distal leg perfusion was secured by inserting a 5 F atrial sheath. All cannulas and sheaths were inserted using the Seldinger technique. The correct initial placement of the cannula tip was verified by ultrasound. The three patients that were initially operated on CPB (either for ASD closure or for LuTx), were all switched to VA-ECMO with peripheral cannulation for post-LuTx VA-ECMO support. Only patient #5 stayed on central cannulation due to his low body height and weight to prevent irreversible occlusive injury to the femoral vessels (Table 2). The weaning strategy from VA-ECMO included regular echocardiographic evaluation of biventricular function on ICU admission postoperatively and once daily after post-OP day 5 on VA-ECMO. ECMO weaning was usually started on day 3 after LuTx at the earliest. On the day of transplantation, the ECMO was started with 80% of the calculated full flow (100% CO) and was reduced stepwise by 20%–25% in two to three steps before explantation. The reduction of ECMO flow was done under direct echocardiographic imaging with a focus on left heart structures to visualize any decrease in LV function, an increase of LV end-diastolic diameter (LVEDD), or potential mitral valve regurgitation (Figure 1). If necessary, further measurements can include Tissue Doppler Imaging (TDI) of the left ventricle and mitral annular plane systolic excursion (MAPSE). Additionally, vital signs, arteriovenous oxygen difference (AVDO_2_), and arterial blood gas analysis (PaO_2_, PaCO_2_) were monitored and a chest-X ray was performed between 12 and 24 h after the reduction step or earlier in case of clinical signs of impaired lung function. All patients were extubated whilst on ECMO support (→awake-VA-ECMO) to avoid pressure and shear stress on the transplanted lungs. Even patient #5, who was small in size and had open chest cannulation, was extubated on day 2 and supported by awake VA-ECMO. Mean and median mechanical ventilation time was 41 and 28 h, respectively (range 17–145 h) (Figure 2, Table 3). In all patients, removal of cannulas was pursued in the operating room and – if necessary – vessels were reconstructed by vascular surgeons. Standard immunosuppression consisted of a combination of oral tacrolimus, mycophenolate-mofetil, and prednisolone (Table 4). No induction therapy was used in our center.

**Table 4:** Dosing regimen for immunosuppression in pediatric lung transplantation (Hannover).

Intraoperatively			
**Methylprednisolone**	**Prednisolone**	**MMF**	**Tacrolimus**
20 mg/kg (max. 1 g) iv	–	–	Start with continuous iv infusion: 0.02 mg/kg/d
Postoperatively, days 0–2			
**Methylprednisolone**	**Prednisolone**	**MMF**	**Tacrolimus**
12 h post OP: 2 mg/kg iv 24 h post OP: 2 mg/kg iv 36 h post OP: 2 mg/kg iv	–	30 mg/kg/d	^a^
Postoperatively, from day 3			
**Methylprednisolone**	**Prednisolone**	**MMF**	**Tacrolimus**
–	d3–d5: 2 mg/kg/d d6–d9: 1 mg/kg/d d10–d28: 0.5 mg/kg/d mo 2: 0.35 mg/kg/d mo 3: 0.25 mg/kg/d mo 4: 0.2 mg/kg/d mo 7: 0.15 mg/kg/d mo 13: 0,1 mg/kg/d	**Target dose:** 1,2 g/m^2^/d **Target drug level:** 1,2–3,5 mg/L	**Target drug levels: months 0–6:** 12–15 µg/L **months 7–12:** 10–12 µg/L **from month 13:** 8–10 µg/L

The standard immunosuppression in pediatric lung transplantation used in our center consisted of oral tacrolimus, mycophenolate-mofetil, and prednisolone. No induction is used in our center.

d, days postoperatively (day 0 = day of surgery); iv, intravenous; MMF, mycophenolate-mofetil; d, day; iv, intravenous.

^a^ Initially, the *tacrolimus dose* is increased very cautiously for nephroprotection. Accordingly, the tacrolimus serum level on postoperative day 2 is usually not in the primary target range of 12–15 µg/L. When continuous intravenous administration is switched to oral tacrolimus, the current cumulative intravenous daily dose is tripled for oral dosing in CF patients and doubled in non-CF patients. The cumulative daily oral dose (divided into two single doses) is then adjusted depending on blood levels.

### 3.6. Adverse events pre-, intra- and post-LuTx

We observed five moderate to severe ECMO-related complications. One intrathoracic hematoma due to dislocation of ECMO cannula pre-LuTx (patient #1), one thromboembolic occlusion of the right iliac/femoral artery due to cannula-related impairment of distal leg perfusion (patient #2), one hemothorax on ECMO requiring thoracotomy on post-LuTx day 1 (patient #3), one intrathoracic hematoma on day 1 post-LuTx requiring surgical removal plus a subsequent thromboembolic left sided subtotal media infarction on day 5 of post-LuTx-ECMO support, with only mild residual neurological deficit (patient #5, who had a prothrombin gene mutation), and one leg ischemia after ECMO-explantation requiring femoral arterial embolectomy (patient #11) (Table 2). Details can be found in the Supplementary Material.

### 3.7. Echocardiographic analysis at baseline and one-year follow-up

**Figure 3:**
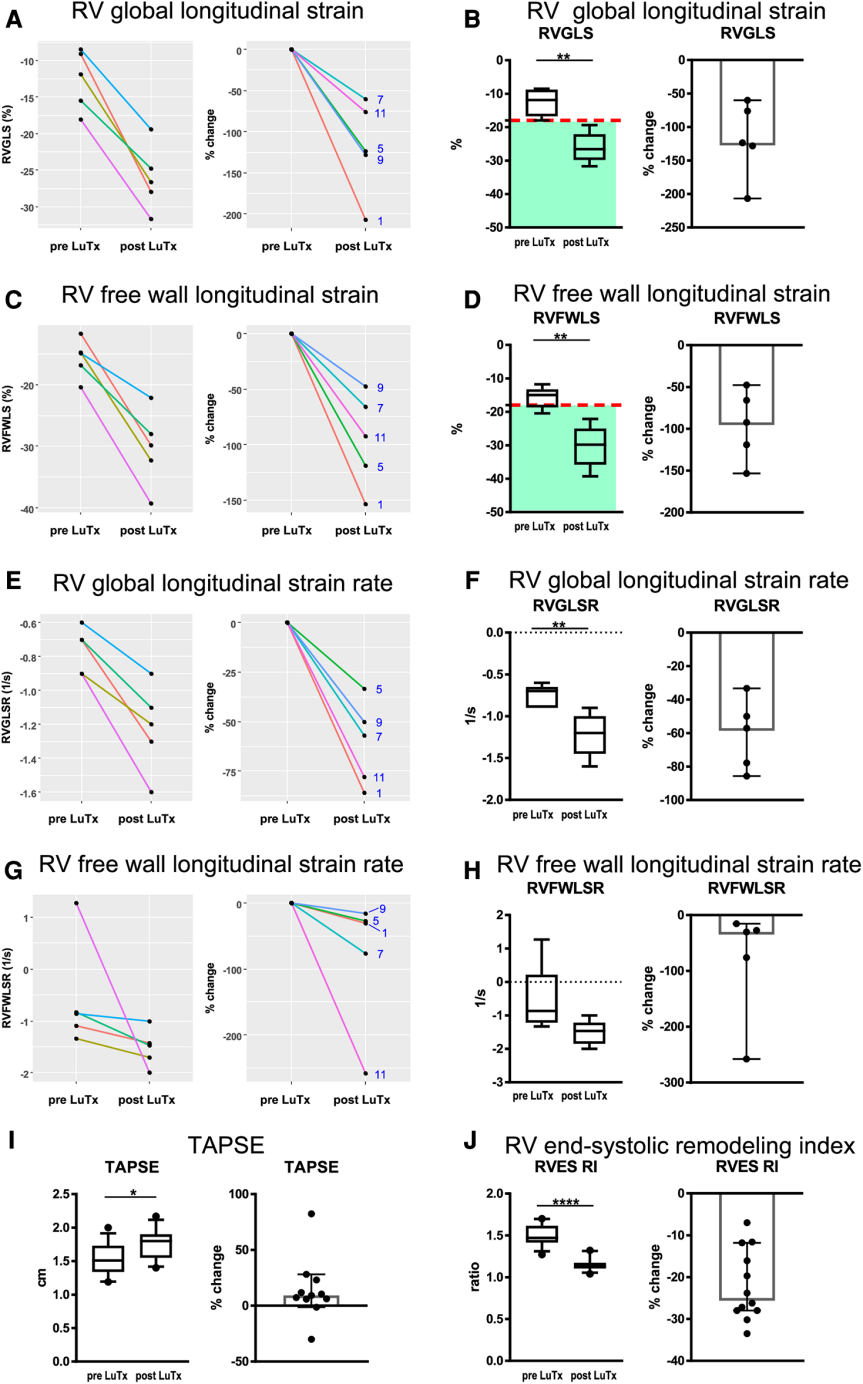
Results of right ventricular strain and strain rate, as well as TAPSE and RV end-systolic remodeling index analysis pre- and post-LuTx. The time points of echocardiography were prior to LuTx (range 0–75 days) and approximately 12 months (range 11–29 months) post-LuTx. The paired two-tailed t-test was used. *p < 0.05; **p < 0.01; ****p < 0.0001, *n* = 5 (**A–H**), *n* = 12 (**I,J**). (**A, C, E, G**) show the individual changes of each patient pre and post-LuTx. The box and whisker plots (third column) show the median, IQR, and 10–90th percentile. The scatter plots (fourth column) show the 95% confidence interval for the median. RV, right ventricle; RV 4CSL, RV 4-chamber longitudinal strain; RVES RI, right ventricular end-systolic remodeling index; TAPSE, tricuspid annular plane systolic excursion.

Transthoracic echocardiography 12 months after transplantation showed full and sustained recovery of RV systolic function in all 12 children after bilateral LuTx, in association with regression of RV hypertrophy (RVH), and normalization of RV volumes (Supplementary Table S4), even in children with severe RV failure pre-LuTx (RVEF < 40%). Mean RVAWD decreased from 1.12 ± 0.10 cm to 0.54 ± 0.05 cm (−50.8% ± 3.6%; Supplementary Table S4), illustrating substantial regression of RV hypertrophy within 12 months. The RV/LV end-systolic diameter ratio as a surrogate of RV dilation and LV underfilling/compression also completely normalized (from 2.38 ± 0.2 to 0.69 ± 0.03, Supplementary Table S4). RV global longitudinal strain (Figures 3A,B), RV free wall strain (Figures 3C,D), and RV global longitudinal strain rate (Figures 3E,F) were greatly abnormal in the PAH patients pre-LuTx (10) and completely normalized 12 months after LuTx (there was likewise a trend for the RV free wall longitudinal strain rate, Figures 3G,H). TAPSE (Figure 3I), as a surrogate for longitudinal systolic RV function, and the RV end-systolic remodeling index (RVES RI) (Figure 3J) were both abnormal pre-LuTx but normalized by the 12-month follow-up.

### 3.8. Clinical follow-up, lung function, and survival 1–2 years after LuTx for pediatric PAH

**Figure 4:**
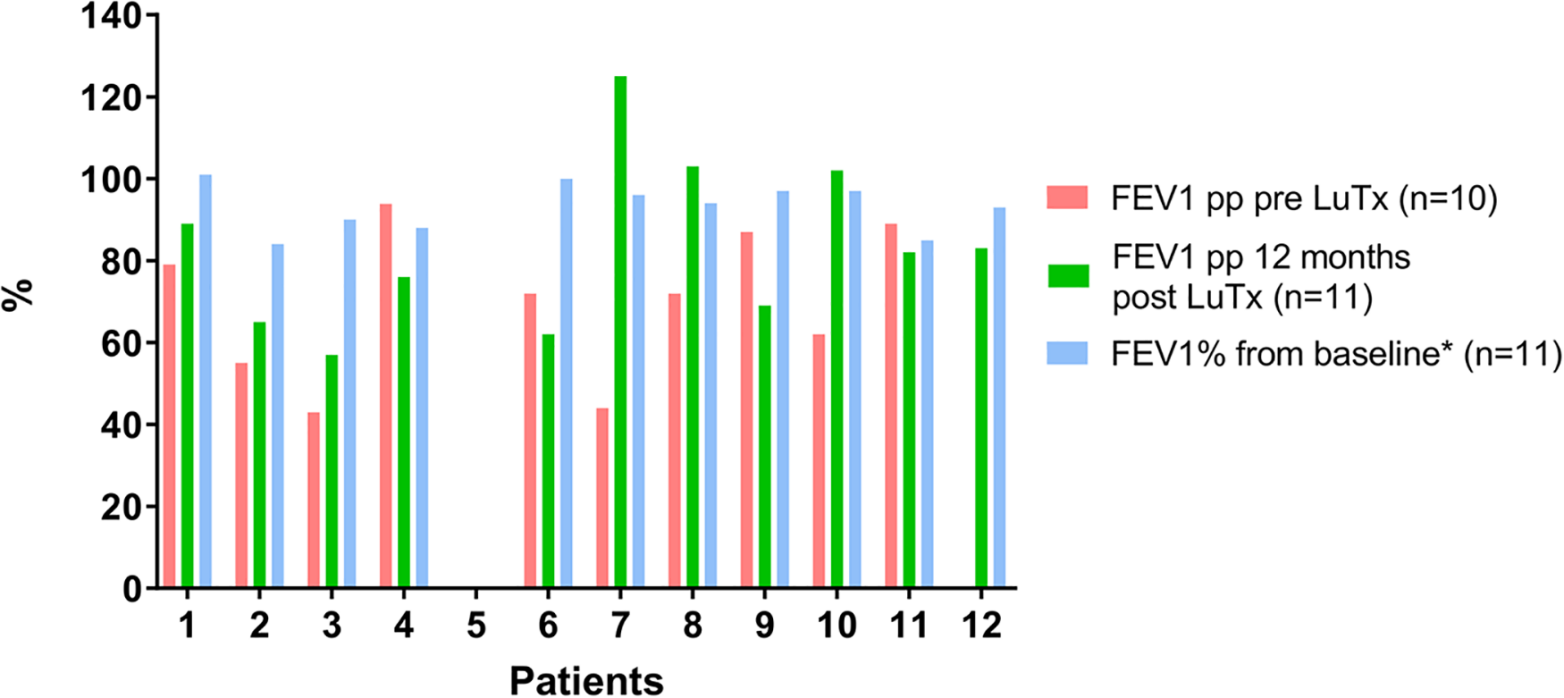
Lung function course of patients with PAH pre- and post-LuTx. Patient #5 was too young to perform spirometry pre-LuTx and during year one post-LuTx. Patient #12 was unable to perform spirometry pre-LuTx. Post-Tx FEV1-Baseline value is computed as the mean of the best two postoperative FEV1 measurements taken >3 months apart to define CLAD starting >3 months post-LuTx. FEV1 >80% of baseline defines the stage CLAD0 (Verleden et al. J Heart Lung Transplant. 2019; 38:493–503. doi: 10.1016/j.healun.2019.03.009) (19). FEV1, forced expiratory volume in 1 s; LuTx, lung transplantation; pp, percent predicted [reference values are taken from Quanjer PH et al. Eur Respir J. 2012 Dec;40(6):1324–43. doi: 10.1183/09031936.00080312] (37).

In the 8-year study period, no patients with PAH died on the LuTx waiting list or during evaluation for LuTx at our center. As of September 1, 2022, all transplanted patients are alive (median survival 53 months, range 26–104 months). Pulmonary function testing pre-LuTx and 12 months post-LuTx are shown in Figure 4. Two children were not able to perform the spirometry maneuver before LuTx, and one of them did not perform the maneuver soon after LuTx due to his/her young age and reduced coordination. Pulmonary function remained stable in all 11 patients able to perform spirometry 12 months post-LuTx (Figure 4). One patient developed CLAD 3 in the third year after LuTx (i.e., after the 2-year follow-up), and was re-transplanted 31 months after the first LuTx. Currently, another patient fulfills the criteria of CLAD 1 (first diagnosed 29 months post-LuTx, i.e. after the 2-year follow-up). Taken together, the clinical follow-up reveals that all 12 patients enrolled are alive 26 months post-LuTx (range 26–104 months).

## 4. Discussion

This prospective observational study illustrates our experience with bilateral lung transplantation and default postoperative VA-ECMO support in children with severe PAH at a single high-volume center. We successfully transplanted all patients according to our standardized VA-ECMO protocol, and all patients were alive after a median follow-up of 53 months (range 26–104 months). Given the low perioperative morbidity, the rapid recovery of RV systolic function after LuTx within 2 months, as shown in our recently published study (10), and the excellent midterm outcome after LuTx for PAH with preserved heart-lung function at one-year follow-up (shown in the current study), LuTx currently appears to be the best and most feasible treatment option for end-stage PAH (group 1 PH), in the absence of complex congenital heart disease. Of the 107 pediatric LuTx patients in the ISHLT registry reported worldwide for 2016, 72% were at least 11 years old, and only six LuTx were performed in children under the age of 1 year (infant LuTx) (9). Overall, LuTx for any condition in children under 12 years is challenging, but outcomes are comparable to those in older children at our center (5), in accordance with ISHLT registry data on children with PVD after LuTx (22). The present and our recently published study (10) showed that even small PH children, below 10 kg body weight, successfully underwent bilateral LuTx with full recovery of heart and lung function. Of note, about a decade ago (2009), we had preferred heart-lung transplantation over bilateral LuTx because of technical aspects for this young age group (23). Today, the remaining conditions for which combined heart-lung transplantation rather than LuTx may be considered, are (1) complex CHD (mostly adults with congenital heart disease and Eisenmenger's syndrome), (2) postcapillary PH due to persistent severe LV dysfunction (e.g., restrictive cardiomyopathy), (3) precapillary PH and additional LV dysfunction that cannot be explained by PAH-related ventricular-ventricular interaction, (4) after intracardiac surgical correction of CHD with complex residual cardiac anatomy, or (5) in patients after surgical correction of multiple pulmonary vein stenoses.

Different aspects possibly hamper further treatment optimization of patients with severe PAH being evaluated for LuTx. Although improvements in waiting time, mortality, and post-transplant survival have occurred in children after the implementation of the lung allocation score (LAS) for LuTx listing of ≥12-year adolescents (24), we found the LAS unsuitable for accurately assessing clinical compromise in children with severe PAH. Due to the different pathophysiology, often without relevant compromised oxygenation and decarboxylation, the LAS underestimates the severity and disease progression in children with PAH as a reason for listing for LuTx (2, 17, 25, 26).

The underestimation of risk is illustrated by the three PAH patients in need of rescue VA-ECMO cannulation before LuTx shortly after referral to our center. The sudden deterioration and RV failure in these three patients underlines the difficulties in determining the optimal timing of listing treatment-resistant PAH patients for LuTx. Indeed, pediatric candidates with severe PH are referred relatively late for LuTx or HLTx transplantation, often requiring immediate intensive care treatment or even veno-arterial ECMO support (2, 5, 12, 25, 26). Moving forward, the EPPVDN pediatric PH risk score (online calculator; https://www.pvdnetwork.org/pedphriskscore/) can properly and quantitatively determine disease severity and suggest listing children with PH for LuTx (18). Additionally, the new lung Composite Allocation Score (lung CAS), which was recently implemented in the United States, includes specific criteria for lung transplant candidates with pulmonary hypertension that can lead to an increase in their waitlist survival and/or post-transplant outcomes scores. The criteria are (1) deteriorating on optimal therapy, and (2) right atrial pressure greater than 15 mmHg or a cardiac index less than 1.8 L/min/m^2^ (https://unos.org/news/lung-cas-score-summary/, accessed on May 31, 2023). Of note, these cut-off criteria are likely not directly applicable to children with PAH who usually have lower RA pressure and higher cardiac index than adults with similar disease severity (see Table 1). The LAS system has not been updated in the Eurotransplant countries since 2011 and there is no scheduled time point in the near future for implementation of the new CAS system. Therefore, comparison with North America regarding the change in waiting time, transplant rates, and survival specifically for PH patients below 12 and between 12 and 18 years of age, whose disease severity is not well represented with the LAS system (still used in Germany), will be very interesting and might influence future modifications of allocation algorithms.

Reverse Potts shunt (27–30), with VA-ECMO backup, might be the only palliative alternative to LuTx for treatment-resistant PAH that may improve morbidity and short-/mid- term survival. In a recent retrospective analysis of the International Potts Shunt Registry, the overall 1- and 5-year transplant-free survival was 77% and 58%, respectively, and 92% and 68% for those discharged home (29). Clearly, the peri-procedural mortality was unacceptably high (17 of 110; 15%) (29) but may improve in high-volume Potts shunt centers with experience, as is the case for LuTx centers. Moreover, establishing a reverse Potts shunt is not applicable for all patients with PAH; only children with systemic or mildly suprasystemic RV pressure and only mildly decreased systolic RV function seem to be suitable candidates for the procedure (29, 31). Children with severely decompensated disease requiring aggressive intensive care are not good candidates for the Potts shunt procedure (29). The group in St. Louis compared the outcome of their pediatric PAH patients after reverse Potts shunt (*n* = 23; 2013-present) and LuTx (*n* = 31; 1995-present) (31). The authors emphasized the lower numbers of peri-procedural complications and shorter ventilation times as positive aspects of the Potts shunt procedure (31). Median ventilation time after LuTx in their cohort was 10.2 days (31) compared to 1.2 days (28 h) in our PAH cohort, highlighting the potential benefits of our default awake VA-ECMO approach that facilitates early extubation.

Compared to the Potts Shunt registry data and other reports on LuTx in pediatric subgroups (25, 29, 32), we experienced only a few severe complications post-LuTx with no or only mild impact on long-term clinical outcomes. Testing all patients in our cohort for acquired von Willebrand syndrome (AVWS), known to increase bleeding risk in moderate to severe PAH (33), and prophylactic von Willebrand factor (VWF)-containing concentrate supplementation (in the presence of AVWS), may have contributed to the low rate of bleeding complications.

The ISHLT Thoracic Transplant registry reported a total of 178 pediatric LuTx for IPAH and 78 pediatric LuTx for PH-non IPAH for the period 2000–2017 (9). The combined PH group (WSPH PH groups 1–5) was the most frequent indication for LuTx in children 0–5 years old (9). According to the ISHLT Registry (32), children with IPAH had the best post-LuTx survival (median 7.4 years) compared to other diagnoses, while those with non-IPAH PH (“secondary PH”) had the highest mortality in the first 10 years after LuTx (median survival 3.2 years) (6). During our 9-year study period (2013–2022), no patient with PAH (judged to be a LuTx candidate) died during LuTx evaluation, on the LuTx-waiting list, or after LuTx at our center that has dedicated pediatric PH and LuTx programs. In contrast, of the five patients who had received LuTx for either WSPH group 2–5 PH or non-PH lung disease at our center, three died (60%) during the same time period post-LuTx, presumably due to the more complex comorbidities (diaphragmatic palsy, chest deformities, upper airway disease, severe cachexia, and severe neuro-developmental disorder), which are associated with a complicated perioperative course. Thus, also for group 2–5 (mainly group 3) PH patients, we suggest referring these children earlier for LuTx evaluation.

LV diastolic dysfunction is common in pediatric and adult PH (1, 34) and has been investigated in a prospective pediatric ventricular function study (35). According to this study, children with PH had LV diastolic dysfunction most consistent with impaired LV relaxation and decreased myocardial deformation, related to invasive hemodynamics, leftward septal shift, and prolonged RV systole. The pre-existing diastolic LV dysfunction in severe PAH is exaggerated directly after LuTx, as a consequence of the greatly increased pulmonary blood flow following the normalization of pulmonary vascular resistance. Consecutively, the increased LV preload post-LuTx frequently leads to pulmonary edema. The latter is addressed by our approach of scheduled post-operative VA-ECMO in all patients undergoing LuTx for PAH (11) to relieve the workload of the LV and protect the transplanted lung from fluid overload and corresponding dysfunction. Default postoperative VA-ECMO generates another relevant benefit after LuTx: even when early ventilatory issues arise post-LuTx, VA-ECMO facilitates early extubation and thus avoids potentially harmful high inspiratory pressures to the allograft soon after LuTx (5, 23). Additionally, there is a risk of primary graft dysfunction as the entire cardiac output is ejected into the allograft by the now unloaded, often hypertrophied RV. The use of VA-ECMO can help mitigate this injury by diverting some of this output to the systemic circulation. Awake-ECMO has been reported for conditions other than PAH associated with heart or lung failure (5, 15, 36). In our center, ECMO patients were extubated as soon as possible. Although early extubation can be challenging in young children, it can be handled by an experienced interdisciplinary team of nurses, physicians, and physiotherapists (15, 16). For awake VA-ECMO, we see clear advantages in reduced sedation/analgetic medication allowing better oral feeding and digestion, better neurological monitoring, and – most importantly for this patient group post-LuTx – early and better airway clearance.

Limitations of this study include the single-center design, the small patient number, and the lack of a control group (for default VA-ECMO). However, our historical controls of LuTx without VA-ECMO or heart-lung transplantation had worse clinical outcomes. Despite these typical limitations in pediatric studies on a rare and fatal disease, our research provides a standardized treatment and weaning protocol that may benefit other centers in the management of pediatric patients with PAH before, during, and after LuTx for PAH.

## 5. Conclusions

In children with severe PAH and RV dysfunction undergoing bilateral LuTx, postoperative VA-ECMO facilitates early extubation with rapid gain of allograft function and also cardiac reverse-remodeling following RV pressure unloading/LV volume loading. Management of PAH children with VA-ECMO after LuTx (and if needed as a bridge to transplantation) results in excellent clinical outcomes, as underpinned by sustained normalization of cardiac performance and preservation of lung allograft function at 1-year follow-up, and 100% survival at 2 years post-LuTx.

## Data Availability

The original contributions presented in the study are included in the article/**Supplementary Material**, further inquiries can be directed to the corresponding author.
