# Supplementary material for: Bilateral lung transplantation for pediatric pulmonary arterial hypertension: perioperative management and one-year follow-up
Source: Front Cardiovasc Med. 2023 Jun 27;10:1193326. doi: 10.3389/fcvm.2023.1193326 (PMC10333590; doi:10.3389/fcvm.2023.1193326)
Supplement: Supplementary file 1 [file Datasheet1.docx]

**Supplementary Material**

**ABBREVIATIONS AND ACRONYMS**

BSA = body surface area

CHD = congenital heart disease

CI = cardiac index, syn. Qsi = systemic blood flow index (Qs indexed to body surface area)

CLAD = chronic lung allograft dysfunction

CPB = cardiopulmonary bypass

HLTx = combined heart and lung transplantation

EPPVDN = European Pediatric Pulmonary Vascular Disease Network

HHT = hereditary hemorrhagic telangiectasia

IPAH/HPAH = idiopathic/heritable pulmonary arterial hypertension

LuTx = lung transplantation

LV = left ventricle

LVEDD = left ventricular end-diastolic diameter

LVEF = left ventricular ejection fraction

LVES EI = left ventricular end-systolic eccentricity index

LVESV = left ventricular end-systolic volume

LVLS = left ventricular longitudinal strain

mRAP = mean right atrial pressure

mPAP = mean pulmonary artery pressure

mSAP = mean systemic artery pressure (aorta)

NTproBNP = N-terminal prohormone of brain natriuretic peptide (NTproBNP)

PAAT = pulmonary artery acceleration time

PAH = pulmonary arterial hypertension

PCH = pulmonary capillary hemangiomatosis

PDA = persistent ductus arteriosus

PH = pulmonary hypertension

PVD = pulmonary vascular disease

PVOD = pulmonary venoocclusive disease

PVRi = pulmonary vascular resistance index (PVR indexed to body surface area)

Qsi = systemic blood flow index (Qp indexed to body surface area), syn. cardiac index

RHF = right heart failure

RV = right ventricle

RVAWD = right ventricular wall diameter (in diastole)

RVEDD = right ventricular end-diastolic diameter

RVH = right ventricular hypertrophy

RV/LV end-systolic ratio = ratios of inner diameters of RV over LV in end-systole

RV mass index = right ventricular mass index

RVEDP = right ventricular end-diastolic pressure

RVEDV index = right ventricular end-diastolic volume (indexed to body surface area)

RVEF = right ventricular ejection fraction

RVES RI = right ventricular end-systolic remodeling index

RVLS/RVRS = right ventricular longitudinal strain/right ventricular radial strain

RVCS = right ventricular circumferential strain

RVCSR = right ventricular circumferential strain rate

S/D ratio = systolic/diastolic duration ratio, CW Doppler flow of tricuspid regurgitation flow

SVRi = systemic vascular resistance (SVR indexed to body surface area)

TAPSE = tricuspid annular plane systolic excursion

TPG = transpulmonary pressure gradient

TR = tricuspid regurgitation

TRV = tricuspid regurgitation velocity (m/s)

SVR = systemic vascular resistance

VA-ECMO = veno-arterial extracorporeal membrane oxygenation

**SUPPLEMENTARY TEXT**

**Supplementary Introduction**

After transplantation, ECMO is commonly used as an emergency treatment in case of severe primary graft dysfunction (PGD) also to minimize additional negative effects of mechanical ventilation on graft function^1^. The need for prolonged mechanical ventilation of the allograft with high PEEP and high driving pressure is associated with worse outcome^2^ and may lead to long-term impairment of lung function, associated with the development of chronic lung allograft dysfunction (CLAD) and the need for re-transplantation.

In addition to enabling early extubation and mobilization after LuTx^3^, the implementation of scheduled and standardized veno-arterial ECMO treatment also allows gradual left ventricular conditioning, preventing LV-congestion and consecutive lung edema, protecting the patient from severe primary graft dysfunction (PGD)^3^.

**Supplementary Methods**

The World Symposium on Pulmonary Hypertension (WSPH, 2018) assigned PH patients with predominant pulmonary veno-occlusive disease (PVOD) or pulmonary capillary hemangiomatosis (PCH) to group 1.6 PH, entitled “PAH with overt features of venous/capillaries (PVOD/PCH) involvement” (see Table S1; Updated PH Classification, WSPH, Nice, 2018). A slightly modified PH classification was published in the 2022 ESC/ERS Guidelines for the diagnosis and treatment of pulmonary hypertension^4^.

**Patient population**

We excluded a 2-year-old female patient with IPAH and a large ASD II from the analysis. It was postulated before LuTx that she was not in need of any postoperative VA-ECMO support because of sufficient LV-pre-load and function via her chronic right-to-left interatrial (large ASD II). The girl is alive and doing well, in WHO functional class (FC) 1, more than 5 years post-LuTx. We also excluded one 11-year-old female who underwent re-transplantation 9 months after initial LuTx due to allograft failure, who is doing well more than 2 years after Re-LuTx as of September 2022.

After June 2020, we transplanted four more PAH patients who are alive and in WHO FC 1 as of September 2022. These four patients had either no sufficient follow-up period at the time point of data acquisition in June 2021 or mixed group 1+3 PH without a confirmed PAH mutation that would qualify the patient(s) to have HPAH (one 3-year-old-girl with HPAH/lung disease; two 6-year-old girls, one with IPAH, one with PAH/lung disease; one 15-year-old girl with PAH-CHD/lung disease).

**Clinical Data Collection**

Pre-operative data included demographics, medical treatment, World Health Organization Functional Class (WHO FC), invasive hemodynamics (mRAP, RVEDP, PAP, etc.), 6-minute walk tests, lung function (FEV1, FVC), NT-proBNP, echocardiography and the EPPVDN risk score.

Perioperative data included VA-ECMO duration pre- and post-Tx, intraoperative MCS-status, mechanical ventilation time, one-month mortality, post-LuTx ICU stay and post-LuTx in-hospital stay. Postoperative and follow-up data included functional status, echocardiography, lung function, survival rate and BOS-free survival.

**Immunosuppression**

Acute cellular rejection (diagnosed by transbronchial biopsies or clinically) is treated with intravenous methylprednisolone (15mg/kg i.v. for three consecutive days). Our treatment protocol for de novo-donor specific antibodies has been published elsewhere^5^.

**Supplementary Results**

**Adverse Events Pre-, Intra- and Post-LuTx**

We observed five severe ECMO-associated complications. Patient #1 developed pre-LuTx an intrathoracic hematoma caused by dislocation of the arterial ECMO cannula inserted under mild sedation in Seldinger technique on the PICU during ECMO-CPR. Immediate intubation and thoracotomy were necessary. Our surgeons changed the cannula position and removed the hematoma. The patient was extubated on the same day and successfully transplanted without any further problems. Patient #2 developed dysfunction of the distal leg perfusion sheath with consecutive thromboembolic occlusion of the right illiac artery/femoral artery on day 2. Embolectomy and successful reconstruction of the vessel was done on ECMO and no further problems occurred in long-term follow-up. Patient #3 required re-thoracotomy on ECMO for hematothorax on day 1 post-LuTx, hematoma was surgically removed and no further bleeding complication occurred. Patient #5 also experienced intrathoracic bleeding with need for surgical intervention on day 1 post-LuTx. This boy had a thrombotic occlusion of the right pulmonary artery in utero due to heterozygous prothrombin gene mutation. Despite strict anticoagulation management, he developed a left sided subtotal media infarction on day 5 on post-LuTx-ECMO support, most likely due a thromboembolic event. He fully recovered and showed only mild residual neurological deficits and no lower limb issues in the follow up examinations. After ECMO explantation, patient #11 was found to have thromboembolic leg ischemia requiring two surgical embolectomies from the right superficial femoral artery post-LuTx, without any clinical sequelae. Four patients had von Willebrand disease type 2 (VWS) and received VWF-containing concentrate (Factor VIII+vWF) supplementation during VA-ECMO pre- and post-LuTx to prevent any bleeding complications. No general bleeding susceptibility was apparent in this subgroup of patients under the preemptive coagulation management.

**Echocardiographic Analysis at Baseline and 1-year Follow-up**

We applied echocardiographic B-mode, M-Mode, Doppler and ventricular strain analysis^6,7^. All examinations were performed on Philipps IE33 or EPIQ CVx ultrasound machines. Images were recorded digitally and analyzed at a workstation using Intellispace Echo software (Philips Medical Systems, The Netherlands) by a single investigator. To assess cardiac remodeling and function pre- and post-LuTx, we determined at least 10 conventional and 2D-speckle tracking echocardiographic variables. For strain analysis, the TomTec RV/LV-AutoStrain / 2D cardiovascular software was used (TomTec Imaging Systems 2.41.00, Unterschleissheim, Germany). Every measurement was thoroughly checked by two independent investigators. The time points of echocardiography were *(1)* prior to LuTx (range 0-75 days), *(2)* an average of 6 weeks (range 1-11 weeks) post-LuTx, and *(3)* approx. 12 months (range 11-29 months) after LuTx.

Due to reduced image quality post-LuTx, LV strain analysis could be performed in only 5 patients. Mean LV longitudinal strain (LV 4CSL) was low normal in the 5 children with PAH pre-LuTx (-21.78% ± 2.72%) and increased to higher values in approximately half of the PAH patients one year post LuTx (25.39% ± 2.42%). Improvement in conventional and advanced echocardiographic parameters of RV function is shown in Table S4.

*Diastolic biventricular function analysis.* Echocardiographic surrogates of diastolic function were difficult to assess because of the low quality of echocardiography after bilateral LuTx. Due to missing data and/or poor quality of the assessment we renounce to display these items.

**Supplementary DISCUSSION**

LV diastolic dysfunction is common in pediatric and adult PH^8,9^ and has been investigated in a prospective, ventricular function study, combining conventional (B-mode, Doppler), biventricular deformation imaging and tissue. Doppler imaging and near-simultaneous cardiac catheterization in 54 pediatric PH patients and 54 matched controls ^10^.

Awake-ECMO in non-intubated pediatric patients has been reported for other indications, such as bridge to recovery in acute lung failure or as the primary rescue method and bridge to destination therapy in heart failure^11-13^. In our center, ECMO patients are extubated as soon as possible. Although early extubation can be challenging in young children, it can be handled by an experienced interdisciplinary team of nurses, physicians and physiotherapists^13,14^. For awake VA-ECMO, we see clear advantages in reduced sedation/analgetic medication allowing better oral feeding and digestion, better neurological monitoring, and - most importantly for this patient group post LuTx - early and better airway clearance. Early extubation also allows mobilization and the possibility of active and intensive physiotherapy to avoid muscle wasting. Moreover, patients in need of emergency cannulation prior to LuTx benefit from this approach and were extubated early after rescue cannulation. The aforementioned advantages of awake VA-ECMO may have contributed to better pre- and post-transplant condition.

**SUPPLEMENTARY TABLES**

**Table S1. Classification of Pulmonary Hypertension (6th World Symposium on Pulmonary Hypertension, Nice 2018)**

| **Group 1-5 Pulmonary Hypertension** |  |
| --- | --- |
| **1. Pulmonary arterial hypertension (PAH)** |  |
| - 1. Idiopathic PAH |  |
| 1.2 Heritable PAH | Causal gene mutations, e.g. BMPR2, ACVRL1, TBX4, EIF2AK4, ATP13A3, GFD2, SOX17, AQP1, SMAD9, ENG, KCNK3, CAV1, … |
| 1.3 Drug and toxin induced | e.g., amphetamines/ methamphetamines, dasatinib |
| 1.4 Associated with: | 1.4.1 Connective tissue disease  1.4.2 HIV infection  1.4.3 Portal hypertension  1.4.4 Congenital heart disease  1.4.5 Schistosomiasis |
| 1.5 PAH long-term responders to calcium channel blockers | Reduction of mPAP ⩾10 mmHg to reach an absolute value of mPAP ⩽40 mmHg.  Increased or unchanged cardiac output Long-term response to CCBs |
| 1.6 PAH with overt features of venous/capillaries (PVOD/PCH) involvement | Pulmonary function tests (Decreased DLCO (frequently <50%)  Chest HRCT (e.g. septal lines; centrilobular ground-glass opacities/nodules)  Response to PAH therapy (possible pulmonary edema) |
| 1.7 Persistent PH of the newborn syndrome |  |
| **2. Pulmonary hypertension due to left heart disease** | 2.1 PH due to heart failure with preserved LVEF  2.2 PH due to heart failure with reduced LVEF 2.3 Valvular heart disease  2.4 Congenital/acquired cardiovascular conditions leading to post-capillary PH |
| **3. Pulmonary hypertension due to lung diseases and/or hypoxia** | 3.1 Obstructive lung disease  3.2 Restrictive lung disease  3.3 Other lung disease with mixed restrictive/obstructive pattern  3.4 Hypoxia without lung disease  3.5 Developmental lung disorders |
| **4. PH due to pulmonary artery obstructions** | 4.1 Chronic thromboembolic PH  4.2 Other pulmonary artery obstructions |
| **5. Pulmonary hypertension with unclear multifactorial mechanisms** | 5.1 Hematological disorders  5.2 Systemic and metabolic disorders  5.3 Others  5.4 Complex congenital heart disease |

From: Simonneau G et al. Eur Resp J 2019; 53: 1801913; DOI: 10.1183/13993003.01913-2018 ^15^

**Table S2. Individual PAH patient characteristics and medication prior to bilateral lung transplantation (LuTx)**

| **No** | **Age (years)** | **Gender**  **(M/F)** | **Weight**  **(kg)** | **BSA (m²)** | **WHOFC**  **(1-4)** | **Diagnosis** | **Invasive Hemodynamics** | **Pulmonary hypertension management pre-LuTx** | **Medical condition pre-LuTx** |
| --- | --- | --- | --- | --- | --- | --- | --- | --- | --- |
| **1** | 15.0 | F | 43.0 (4^th^ Perc.) | 1.40 | 4 | PCH/PAH  (group 1.6 PH) | mRAP: 10 mmHg  RVEDP: 12 mmHg  mPAP/mSAP: 1.11  PVRi: 33.2 WU·m^2^  PVR/SVR: 1.62  Qsi: 1.9 L/min/m^2^ | ILO, SIL, BOS, SPI, FUR, 15 L O_2_/min | Acute RHF, alveolar diffusion impairment, VA-ECMO-CPR pre-LuTx,  22 hours on VA-ECMO pre-LuTx |
| **2** | 13.2 | F | 40.0 (12^nd^ Perc.) | 1.34 | 4 | IPAH  (group 1.1 PH),  type 1 diabetes | mRAP: 11 mmHg  RVEDP: 13 mmHg  mPAP/mSAP: 1.19  PVRi: 23.9 WU·m^2^  PVR/SVR: 1.41  Qsi: 2.3 L/min/m^2^ | EPIV, SIL, BOS, FUR | Acute RHF, ECMO-CPR pre-LuTx, 292 hours on ECMO pre-LuTx |
| **3** | 10.7 | M | 35.0  (42^nd^ Perc.) | 1.27 | 4 | IPAH  (group 1.1 PH) | mRAP: 17 mmHg  RVEDP: 19 mmHg  mPAP/mSAP: 1.10  PVRi: 24.3 WU·m^2^  PVR/SVR: 1.33  Qsi: 2.3 L/min/m^2^ | TREP i.v., SIL, MAC, SPI, ASA | Progression of PH, RHF |
| **4** | 14.2 | F | 50.0  (34^th^ Perc.) | 1.53 | 4 | PAH-CHD  (group 1.4.4 PH)  s/p d-TGA repair, vWS type 2 | mRAP: 15 mmHg  RVEDP: 15 mmHg  mPAP/mSAP: 1.52  PVRi: 29.7 WU·m^2^  PVR/SVR: 2.69  Qsi: 2.8 L/min/m^2^ | TREP i.v., RIO, BOS, 2 L O2/min | Progression of PH, acute RHF |
| **5** | 1.9 | M | 8.2  (< 1^st^ Perc.) | 0.42 | 3-4 | PAH-CHD (group 1.4.4 PH), Preterm 29 + 2 GW, IRDS, prothrombin mutation, severe RPA hypoplasia, ASD II, s/p PDA closure | Last cath > 12 months  pre-LuTx | SIL, MAC, CLO, SPI, 0.75 L O_2_/min | Progression of PH, RHF |
| **No** | **Age (years)** | **Gender**  **(M/F)** | **Weight**  **(kg)** | **BSA (m²)** | **WHOFC**  **(1-4)** | **Diagnosis** | **Invasive Hemodynamics** | **Pulmonary hypertension management pre-LuTx** | **Medical condition pre-LuTx** |
| **6** | 17.5 | F | 40.0  (< 1^st^ Perc.) | 1.32 | 3 | HPAH, BMPR2 mutation  (group 1.2 PH),  M. Osler (HHT), small PFO | mRAP: 4 mmHg  RVEDP: 9 mmHg  mPAP/mSAP: 1.13  PVRi: 38.1 WU·m^2^  PVR/SVR: 1.24  Qsi: 2.7 L/min/m^2^ | ILO inhal., SIL, MAC, SPI | Progression of PH |
| **7** | 10.3 | F | 25.0  (3^rd^ Perc.) | 0.92 | 4 | HPAH, TBX4 mutation  (group 1.2 PH),  IRDS, chILD, PFO, small patella syndrome | mRAP: 4 mmHg  RVEDP: 11 mmHg  mPAP/mSAP: 1.22  PVRi: 36.1 WU·m^2^  PVR/SVR: 0.99  Qsi: 2.4 L/min/m^2^ | ILO inhal., SIL, MAC, SPI, 1.5-2.5 L O_2_/min | Progression of PH |
| **8** | 11.7 | M | 33.0  (15^th^ Perc.) | 1.15 | 4 | PVOD/PAH  (group 1.6 PH),  Preterm 32 + 5 GW, s/p gastroschisis, double aortic arch with atresia of the left arch, type 2 vWD, intervent. rASD 09/2017 | mRAP: 5 mmHg  RVEDP: 11 mmHg  mPAP/mSAP: 1.18  PVRi: 22.3 WU·m^2^  PVR/SVR: 1.16  Qsi: 3.4 L/min/m^2^ | TREP i.v., TAD, BOS, AML, SPI, ASA | Progression of PH, acute RHF, VA-ECMO-CPR pre-LuTx, 46 hours on VA-ECMO pre-LuTx |
| **9** | 17.8 | F | 58.0  (46^th^ Perc.) | 1.68 | 3 | IPAH  (group 1.1 PH),  type 2 vWD, migraine | mRAP: 10 mmHg  RVEDP: N/A  mPAP/mSAP: 1.10  PVRi: 36.8 WU·m^2^  PVR/SVR: 1.06  Qsi: 2.3 L/min/m^2^ | TREP i.v. infusion pump, SIL, MAC, DIG, SPI | Progression of PH |
| **10** | 16.2 | F | 52.0  (24^th^ Perc.) | 1.54 | 4 | IPAH  (group 1.1 PH), intervent. rASD 01/2019 | mRAP: 8 mmHg  RVEDP: 14 mmHg  mPAP/mSAP: 0.88  PVRi: 24.2 WU·m^2^  PVR/SVR: 0.91  Qsi: 2.4 L/min/m^2^ | ILO inhal., SIL, MAC, AML, ASA, SPI, 2L O_2_/min | Progression of PH |
| **No** | **Age (years)** | **Gender**  **(M/F)** | **Weight**  **(kg)** | **BSA (m²)** | **WHOFC**  **(1-4)** | **Diagnosis** | **Invasive Hemodynamics** | **Pulmonary hypertension management pre-LuTx** | **Medical condition pre-LuTx** |
| **11** | 5.5 | F | 16.8  (9^th^ Perc.) | 0.73 | 3 | HPAH, BMPR2 mutation  (group 1.2 PH) | mRAP: 10 mmHg  RVEDP: 14 mmHg  mPAP/mSAP: 1.29  PVRi: 13.9 WU·m^2^  PVR/SVR: 1.61  Qsi: 4.1 L/min/m^2^ | EPIV, SIL, BOS, AML | Progression of PH, RHF |
| **12** | 8.2 | F | 21.8  (7^th^ Perc.) | 0.89 | 3-4 | HPAH, BMPR2 mutation  (group 1.2 PH), s/p VSD repair, type 2 vWD | mRAP: 9 mmHg  RVEDP: 9 mmHg  mPAP/mSAP: 1.17  PVRi: 17.8 WU·m^2^  PVR/SVR: 1.58  Qsi: 2.9 L/min/m^2^ | Levosimendan (repetitive), SIL, MAC, SEL, SPI | Progression of PH, acute RHF |

**Table S2.** **Individual PAH patient characteristics and medication prior to bilateral lung transplantation (LuTx).** Invasive hemodynamics are the last measurements in the preceding 12 months before LuTx. Abbreviations: AML, amlodipine; ASA, acetylsalicylic acid (P.O.); BSA, body surface area; CHD, congenital heart disease; CLO, clopidogrel; CPB, cardiopulmonary bypass; CPR, cardiopulmonary resuscitation; digoxin (P.O.); EPIV; epoprostenol i.v.; FUR, furosemide (P.O.); HPAH, hereditary PAH; ILO, iloprost; IPAH, idiopathic PAH; LuTx, lung transplantation; NT-proBNP, N-terminal pro b-type natriuretic peptide; PAH, pulmonary arterial hypertension; PCH, pulmonary capillary hemangiomatosis; PVOD, pulmonary venoocclusive disease; RHF, right heart failure; SEL, selexipag (P.O.); SIL, sildenafil (P.O.); SPI, spironolactone (P.O.); TAD, Tadalafil; TREP, treprostinil; VA-ECMO, veno-arterial extracorporeal membrane oxygenation

**Table S3. Individual patient data related to bilateral lung transplantation for PAH, post-operative course and clinical follow-up (all alive)**

| **No** | **MCS type during LuTx and associated procedures** | **OP time**  **cut-suture**  **(hours)** | **Post-LuTx ventilation time (hours)** | **ICU stay post-LuTx**  **(days)** | **In-hospital stay post-LuTx**  **(days)** | **Lung function post LuTx: FEV1 (%)** | **Survival post-LuTx (months)** |
| --- | --- | --- | --- | --- | --- | --- | --- |
| **1** | VA-ECMO | 5.0 | on-ECMO: 26  after ECMO-explantation: 0 | 16 | 57 | FEV1 3 months post-LuTx: 61%  FEV1 12 months post-LuTx: 89% | 104 |
| **2** | VA-ECMO | 4.6 | on-ECMO: 23  after ECMO-explantation: 0 | 15 | 47 | FEV1 3 months post-LuTx: 57%  FEV1 12 months post-LuTx: 65% | 103 |
| **3** | VA-ECMO | 5.7 | on-ECMO: 24  after ECMO-explantation: 6 | 9 | 41 | FEV1 3 months post-LuTx: 58%  FEV1 12 months post LuTx: 57% | 83 |
| **4** | VA-ECMO | 10.1 | on-ECMO: 41  after ECMO-explantation: 1 | 11 | 27 | FEV1 3 months post-LuTx: 71%  FEV1 12 months post-LuTx: 76% | 78 |
| **5** | VA-ECMO | 9.0 | on-ECMO: 41  after ECMO-explantation: 6 | 32 | 60 | FEV1 3 months post-LuTx: N/A  FEV1 12 months post-LuTx: N/A | 66 |
| **6** | VA-ECMO | 5.6 | on-ECMO: 144  after ECMO-explantation: 1 | 28 | 62 | FEV1 3 months post-LuTx: 52%  FEV1 12 months post-LuTx: 62% | 57 |
| **7** | VA-ECMO | 7.1 | on-ECMO: 29  after ECMO-explantation: 5 | 12 | 30 | FEV1 3 months post-LuTx: 106%  FEV1 12 months post-LuTx: 125% | 49 |
| **8** | ASD closure on CPB, LuTx on VA-ECMO | 6.1 | on-ECMO: 67  after ECMO-explantation: 0 | 23 | 54 | FEV1 3 months post-LuTx: 66%  FEV1 12 months post-LuTx: 103% | 48 |
| **9** | VA-ECMO | 6.5 | on-ECMO: 17  after ECMO-explantation: 0 | 4 | 21 | FEV1 3 months post-LuTx: 67%  FEV1 12 months post-LuTx: 69% | 46 (including Re-LuTx 31 months after initial LuTx) |
| **10** | ASD closure on CPB, LuTx on VA-ECMO | 7.1 | on-ECMO: 20  after ECMO-explantation: 2 | 10 | 26 | FEV1 3 months post-LuTx: 101%  FEV1 12 months post-LuTx: 102% | 41 |
| **11** | CBP, post-LuTx VA-ECMO | 6.5 | on-ECMO: 19  after ECMO-explantation: 0 | 8 | 26 | FEV1 3 months post-LuTx: 76%  FEV1 12 months post-LuTx: 82% | 30 |
| **12** | VA-ECMO | 6.0 | on-ECMO: 25  after ECMO-explantation: 0 | 9 | 35 | FEV1 3 months post-LuTx: 87%  FEV1 12 months post-LuTx: 83% | 26 |

**Table S3. Individual patient data related to bilateral lung transplantation for PAH, post-operative course and clinical follow-up (all alive).** Survival is indicated according to the end of follow-up (September 1, 2022). Abbreviations: ASD, atrial septal defect; CPB, cardiopulmonary bypass; FEV1, forced expiratory volume in the first second; LuTx, lung transplantation; MCS, mechanical circulatory support; VA-ECMO, veno-arterial extracorporeal membrane oxygenation.

**Table S4. Improvement of conventional and advanced echocardiographic parameters of RV function in the 12 patients 1-year post LuTx.**

| **Echocardiographicparameters** | **Number of patients (n)** | **pre LuTx (mean ± SEM)** | **1 yr post LuTx (mean ± SEM)** | **max. % change** | **p-value** |
| --- | --- | --- | --- | --- | --- |
| **RVAWD [cm]** | 12 | 1.12 ± 0.10 | 0.54 ± 0.05 | -50.77 | 0.0002 |
| **TAPSE [cm]** | 11 | 1.54 ± 0.07 | 1.74 ± 0.07 | +13.93 | 0.0488 |
| **RVES RI** | 12 | 1.50 ± 0.04 | 1.15 ± 0.02 | -21.93 | <0.0001 |
| **RV/LV ES diameter ratio** | 11 | 2.38 ± 0.21 | 0.69 ± 0.03 | -71.37 | <0.0001 |
| **LVES EI** | 12 | 2.12 ± 0.15 | 1.11 ± 0.03 | -45.05 | <0.0001 |
| **PAAT [ms]** | 10 | 61.33 ± 3.70 | 134.09 ± 3.36 | +124.28 | <0.0001 |
| **PA VTI [cm²]** | 7 | 13.05 ± 1.27 | 22.16 ± 1.40 | +93.60 | 0.0156 |

**Table S4. Improvement of conventional and advanced echocardiographic parameters of RV function in the 12 patients 1-year post LuTx.** Conventional and advanced echocardiographic variables normalized 1 year after LuTx. Mean ± SEM and the max. % change is given pre and one year post LuTx. Test: Either Wilcoxon matched-pairs signed rank test or paired t-test. Abbreviations: LVES EI, left ventricular end-systolic eccentricity index; PAAT, pulmonary artery acceleration time; PA VTI, pulmonary artery velocity time integral; RVAWD, right ventricle anterior wall diameter; RVES RI, right ventricular end-systolic remodeling index; TAPSE, tricuspid annular plane systolic excursion.

**Supplementary References**

1. Gulack BC, Hirji SA, Hartwig MG. Bridge to lung transplantation and rescue post-transplant: the expanding role of extracorporeal membrane oxygenation. *J Thorac Dis*. 2014;6:1070-1079. doi: 10.3978/j.issn.2072-1439.2014.06.04

2. Paraskeva MA, Borg BM, Paul E, Fuller J, Westall GP, Snell GI. Abnormal one-year post-lung transplant spirometry is a significant predictor of increased mortality and chronic lung allograft dysfunction. *J Heart Lung Transplant*. 2021;40:1649-1657. doi: 10.1016/j.healun.2021.08.003

3. Tudorache I, Sommer W, Kuhn C, Wiesner O, Hadem J, Fuhner T, Ius F, Avsar M, Schwerk N, Bothig D, et al. Lung transplantation for severe pulmonary hypertension--awake extracorporeal membrane oxygenation for postoperative left ventricular remodelling. *Transplantation*. 2015;99:451-458. doi: 10.1097/TP.0000000000000348

4. Humbert M, Kovacs G, Hoeper MM, Badagliacca R, Berger RMF, Brida M, Carlsen J, Coats AJS, Escribano-Subias P, Ferrari P, et al. 2022 ESC/ERS Guidelines for the diagnosis and treatment of pulmonary hypertension. *Eur Heart J*. 2022;43:3618-3731. doi: 10.1093/eurheartj/ehac237

5. Ius F, Sommer W, Tudorache I, Avsar M, Siemeni T, Salman J, Molitoris U, Gras C, Juettner B, Puntigam J, et al. Five-year experience with intraoperative extracorporeal membrane oxygenation in lung transplantation: Indications and midterm results. *J Heart Lung Transplant*. 2016;35:49-58. doi: 10.1016/j.healun.2015.08.016

6. Koestenberger M, Friedberg MK, Ravekes W, Nestaas E, Hansmann G. Non-Invasive Imaging for Congenital Heart Disease: Recent Innovations in Transthoracic Echocardiography. *J Clin Exp Cardiolog*. 2012;Suppl 8:2. doi: 10.4172/2155-9880.S8-002

7. Truong U, Meinel K, Haddad F, Koestenberger M, Carlsen J, Ivy D, Jone PN. Update on noninvasive imaging of right ventricle dysfunction in pulmonary hypertension. *Cardiovasc Diagn Ther*. 2020;10:1604-1624. doi: 10.21037/cdt-20-272

8. Hansmann G. Left ventricular diastolic dysfunction in pediatric pulmonary hypertension. *Circ Cardiovasc Imaging*. 2016;9. doi: 10.1161/CIRCIMAGING.116.005527

9. Hansmann G. Pulmonary hypertension in infants, children, and young adults. *J Am Coll Cardiol*. 2017;69:2551-2569. doi: 10.1016/j.jacc.2017.03.575

10. Burkett DA, Slorach C, Patel SS, Redington AN, Ivy DD, Mertens L, Younoszai AK, Friedberg MK. Impact of Pulmonary Hemodynamics and Ventricular Interdependence on Left Ventricular Diastolic Function in Children with Pulmonary Hypertension. *Circ Cardiovasc Imaging*. 2016;9:pii: e004612. doi: doi: 10.1161/CIRCIMAGING.116.004612.

11. Iablonskii P, Carlens J, Mueller C, Aburahma K, Niehaus A, Boethig D, Franz M, Floethmann K, Sommer W, Optenhoefel J, et al. Indications and outcome after lung transplantation in children under 12 years of age: A 16-year single center experience. *J Heart Lung Transplant*. 2022;41:226-236. doi: 10.1016/j.healun.2021.10.012

12. Ius F, Aburahma K, Boethig D, Salman J, Sommer W, Draeger H, Poyanmehr R, Avsar M, Siemeni T, Bobylev D, et al. Long-term outcomes after intraoperative extracorporeal membrane oxygenation during lung transplantation. *J Heart Lung Transplant*. 2020;39:915-925. doi: 10.1016/j.healun.2020.04.020

13. Schmidt F, Jack T, Sasse M, Kaussen T, Bertram H, Horke A, Seidemann K, Beerbaum P, Koeditz H. "Awake Veno-arterial Extracorporeal Membrane Oxygenation" in Pediatric Cardiogenic Shock: A Single-Center Experience. *Pediatr Cardiol*. 2015;36:1647-1656. doi: 10.1007/s00246-015-1211-8

14. Schmidt F, Sasse M, Boehne M, Mueller C, Bertram H, Kuehn C, Warnecke G, Ono M, Seidemann K, Jack T, et al. Concept of "awake venovenous extracorporeal membrane oxygenation" in pediatric patients awaiting lung transplantation. *Pediatr Transplant*. 2013;17:224-230. doi: 10.1111/petr.12001

15. Simonneau G, Montani D, Celermajer DS, Denton CP, Gatzoulis MA, Krowka M, Williams PG, Souza R. Haemodynamic definitions and updated clinical classification of pulmonary hypertension. *Eur Respir J*. 2019;53. doi: 10.1183/13993003.01913-2018
